# Getting Angry

**Published:** 1873-01

**Authors:** 


### GETTING ANGRY

Never does any good; it always does harm. The generous hearted may compassionate and pity, but they never descend so low as to get mad. Anger debases always, unless there is strength of character enough to conceal it; but the misfortune is, the weakest minded are the most passionate.

If a man ever feels himself utterly contemptible, it is when he has been allowed to give expression to his feelings in the excitement of passion in the presence of ladies and gentlemen; there is a self-inflicted punishment, almost greater than he can bear, and gladly would he hide himself in an auger hole, if possible, or slide away into the darkest corner.

If you want to "heap coals of fire" on the inner heart of one who is in a tearing passion, and is expressing it in words, just simply say nothing; do nothing; only look at him in silence, and it will almost kill him, for he has a consciousness of the fact that every one who has heard him despises him. No one can ever feel at home with a churlish, crusty, ill-natured person, for there is no reliance to be placed on his moods; he may be in the best possible humor one instant, and the next be literally "raving."

There is an instinctive aversion against having anything to do with passionate people; it is unpleasant to have any business engagements with them. We patronize the cheerful, the good hearted man—the man who is willing to accommodate, and is ever ready to do a good turn. In a business point of view, the touchy man is always at a disadvantage; passionateness does not invite custom, pays no debts, makes creditors more exacting and less willing to wait. It was pithily said, three thousand years ago, "Anger dwelleth in the bosom of fools." If you must be angry, simply keep your mouth shut; you will be thankful for it half an hour later, and will certainly feel yourself to be that much more of a man.

There is Miss Snap—she is always pecking at somebody; she has a spiteful word foreverybody; her expletives are always depreciatory; in her eye, the motives of every human being are mean and selfish. But who respects Miss Snap? Has she a friend on earth? No, not one.

